# Photographic scale for the characterization of actinic keratosis through reflectance confocal microscopy: a quantitative approach to cellular transformation

**DOI:** 10.3389/fmed.2024.1391859

**Published:** 2024-09-04

**Authors:** Lucas Offenbecker Guerra, Ana Carolina Leão Santos, Janaina Rosa Cortinoz, Renata Ferreira Magalhães, Louise Idalgo Vasques, Gislaine Ricci Leonardi

**Affiliations:** ^1^Faculty of Medical Sciences, University of Campinas (UNICAMP), Campinas, Brazil; ^2^ALS - Allergisa Pesquisa Dermato-Cosmetica Ltda., Campinas, Brazil; ^3^Faculty of Pharmaceutical Sciences, University of Campinas (UNICAMP), Campinas, Brazil; ^4^Dermatology Outpatient Clinic, Clinics Hospital, University of Campinas (UNICAMP), Campinas, Brazil

**Keywords:** actinic keratosis, cellular transformation, skin cancer, clinical diagnosis, photographic scale, dermoscopy, reflectance confocal microscopy

## Abstract

**Background:**

Actinic keratosis (AK) is a highly prevalent pre-cancerous skin lesion that often leads to cutaneous squamous cell carcinoma. There are different stages of evolution of the disease and several features that characterize keratosis. This study aimed to develop a qualitative and quantitative visual diagnostic tool to facilitate the identification of the characteristics and severity of the main cellular attributes of AK and to show its applicability in evaluating the evolution or treatment through image analysis.

**Methods:**

Literature research on the main scientific databases and in the institute’s database was carried out to gather all the different levels of cellular transformation. To validate the scale, a preliminary characterization study was carried out with 21 subjects who had clinically diagnosed AK lesions to classify the attributes in each skin layer and test the accuracy of the diagnosis of the scale. Afterward, and to show the possibility of a follow-up with a topical treatment, the subjects were divided into two treatment groups, receiving either a cream formulation containing retinoic acid, or a placebo formula. The evaluation was carried out through confocal reflectance microscopy and a digital camera with dermoscopic quality before and after 90 days of treatment.

**Results:**

A table detailing the 18 attributes of AK, and a photographic scale containing RCM images graded by scores established for each characteristic and the frequency of spreading were developed. The results of the validation presented good repeatability, correlation with clinical evaluation, and capacity for differentiating treatments demonstrated by the significant improvement after topical treatment by the reduction of the score for 10 out of the 18 attributes. The preliminary study, evaluated by the detailed transformation scale highlights important differences in the subclinical approach that allows a deeper evaluation of the aspects of the lesion’s re-incidence even after fully treated skin sites.

**Conclusion:**

This study brings an innovative method based on RCM, to assist in the quantification of cell transformation level, provide early diagnosis, and deliver a powerful treatment evaluation tool to provide smoother treatment, as well as prevent re-incidence in the cases.

## Introduction

### Definition, prevalence, and characteristics of actinic keratosis

Actinic keratosis (AK) is one of the most prevalent precancerous lesions in the world, but, due to its low mortality in this stage, the lesions are often neglected, which increases the number of transformation cases into cutaneous squamous cell carcinoma (cSCC). It is estimated that 0.025 to 16% of the cases progress to cSCC, but between 40 and 60% of carcinomas begin because of untreated keratoses (1–3).

Regarding its definition, AK is described as an intraepithelial keratinocytic dysplasia characterized by small, irregular, rough pink, red, or beige skin lesions located on the most exposed areas of the body to the sunlight (face, ears, back of hands, and arms). When it appears on the lips, it is called actinic cheilitis (AC). It can also manifest as a cutaneous horn—hypertrophic actinic keratosis (HAC)—characterized by a hard, dark, or yellowish mass, with a conical shape (4–6).

Although AKs are traditionally classified as pre-neoplastic lesions, some authors consider them as neoplasms *in situ*, since they derive from clonal DNA modifications in keratinocytes (7–11). In this sense, AKs have characteristics of malignancy since their origin, showing cellular changes in epidermal keratinocytes almost indistinguishable from the ones observed in cSCC, such as loss of polarity, nuclear pleomorphism, dysregulated maturation, increased number of mitoses, and molecular alterations, presenting identical mutations in the p53 protein (12–16).

The risk factors for developing AK are variable, however, the most common factors are related to a history of unprotected exposure to ultraviolet (UV) radiation from the sun or indoor tanning, geographic location—increasing with the proximity to the equator—, weakened immune system due to medical conditions or medications, low Fitzpatrick phototype, age over 40 (17, 18).

AK usually does not exist as a single lesion, with clinical and subclinical lesions being able to affect an entire area of sun-exposed skin resulting in field cancerization (19–24), which is a term used to describe areas of skin affected by chronic UV radiation. The affected skin region can develop visible AKs and harbor subclinical AKs. Field cancerization includes both visible AK lesions and invisible subclinical damage and, to effectively treat the entire region, field-directed therapy is required (25–27).

### Diagnose and treatments

AK is diagnosed through clinical examination and visual inspection performed by a trained Dermatologist. Dermoscopy, confocal laser scanning, microscopy, and optical coherence tomography may be used for the diagnosis of AK and cSCC if clinical findings are ambiguous (28). AK does not require histological confirmation as it used to do if the clinical findings are characteristic, especially because invasive methods such as biopsy cannot be performed on large areas of skin, due to the discomfort to the patient and the risk of scarring (29). However, clinically ambiguous lesions, that show signs of progression to cSCC, or whose biological behavior cannot be assessed should be biopsied. Histology shall also be obtained for AK that do not respond to adequate treatment (15). The early diagnosis and treatment of AK can help prevent the appearance of new lesions and can identify early signs of cell transformation into skin cancer, preventing further complications from the disease.

The clinical analysis according to Olsen et al. (30) classifies AK in three degrees 0—slightly visible and palpable; 1—visible and palpable; and 2—frankly visible and hyperkeratotic. AKs cannot always be distinguished from cSCC *in situ* or invasive cSCC (31). This evaluation has a precision of 74 to 94% (11, 32, 33).

The dermoscopic analysis provides a deeper perspective by recognizing three lesion patterns—a red pseudo network pattern with discrete white scales, an erythematous background known as the “strawberry pattern,” and enlarged follicular openings filled with keratotic content over a scaly and white-to-yellow-appearing background (34).

More precise than clinical and dermoscopic analysis is the *in vivo* microscopic examination through reflectance confocal microscopic (RCM). RCM is a clinical diagnostic imaging tool that provides a horizontal scan of the skin at cellular level resolution using different refractive indices and scanning from the epidermal layers and the superficial and medial layers of the dermis to the level of cellular and structural organization of the tissue. It has been widely used in the clinical diagnosis of several skin diseases due to its non-invasive nature, being *in vivo*, presenting a high level of repeatability and the possibility of real-time evaluation (35, 36). Several studies showed image characteristics of RCM on AK, being helpful in the diagnosis of the majority of the cases (37–40).

Regarding treatment, individual AKs are treated with lesion-directed therapy. For multiple AKs located in one area, field-directed therapy is the treatment of choice. Field-directed therapies include the FDA-approved topical agents 5-fluorouracil (5-FU), imiquimod, diclofenac, tirbanibulin, and photodynamic therapy. For all treatments, studies showing both satisfactory results and some adverse reactions can be found, with the 5-FU and imiquimod usually presenting more severe and systemic reactions and the others, presenting milder and local reactions. The most common adverse reactions in the skin are local pain, pruritus, burning sensation, hyperpigmentation, local erythematous inflammation, edema, dryness, flaking/scaling, swelling, crusting, erosions, and ulcerations (15, 41, 42).

Furthermore, topical treatments with retinoids have been extensively reported as successful for various skin pathologies and conditions of the skin such as acne, melasma, and photoaging signs, being well-known and widely used for the prevention and treatment of non-melanoma skin cancers, even though its mechanism is still not completed elucidated (43, 44). Clinical studies evaluated the efficacy and safety of its use and no clear evidence of a relation between the use of topical tretinoin and the development of systemic adverse effects was found (45). However, its use is contraindicated in pregnant women and the use of contraceptive methods in women of childbearing age should be advised (46, 47). Among the adverse effects, skin irritation is one of the most reported; however, over the first 2 or 3 months of treatment, good tolerability has been observed (48, 49).

Given the difficulty in identifying and leveling the subclinical attributes of AK by severity and scattering, and that there are no characterization studies that demonstrate all attributes in a simplified way, the main objective of this study was to create a photographic scale based on RCM images to serve as a didactic tool to assist in early diagnosis, quantitatively identify the different characteristics of cellular transformation in skin lesions and also validate the proposed tool by showing its usability in clinical practice.

## Materials and methods

The photographic scale validation was performed concomitantly to an efficacy preliminary study to assess the efficacy of a topical formulation to treat AK containing 0.1% retinoic acid compared to a placebo product. Retinoic acid is an active ingredient that has been successful in the treatment of many skin conditions, including AK with mild adverse effects. Subjects previously recruited were randomly divided into two groups—the placebo group, whose subjects received a basic formulation without any active ingredient, and the treatment group, whose subjects received a formulation containing 0.1% retinoic acid in a cream base.

Subjects applied the formula at home for 90 days and were assessed by a dermatologist to grade AK level and reflectance microscopy confocal image acquisition in two different time-points at the baseline (T0) and after 90 days of product use (T90), from now assigned as T0 and T90, respectively.

Thus, the study was divided into three parts. The first part consisted of acquiring the dermoscopy and RCM images to construct the scale; the second part consisted of carrying out a preliminary study to validate the scale in diagnosis and repeatability and; the third part consisted of following up on the improvement of the AK lesions treated with a topical treatment.

### Inclusion criteria

For this study, 21 men and women, aged between 50 and 89 years old, who had skin lesions with visual characteristics of AK were recruited and once they had agreed to participate within the study conditions, signed the consent form.

### RCM image acquisition for the actinic keratosis photographic scale

Before starting the skin assessment, the subjects stayed in an air-conditioned room for 15 min and had their skin cleaned. The regions of skin with the greatest interest were photographed with VivaCam^®^ to be used as a guide for acquiring the microscopic images afterward. From the images with dermoscopic quality, the structural and morphological characteristics of the epidermis were evaluated (50).

The evaluation of the cellular characteristics of the different layers of the skin was performed using the Vivascope^®^ 1500 laser reflectance confocal microscope, which uses a laser source with a wavelength of 830 nm and an immersion objective capable of detecting 20 images per second (51). The microscopic images of 5 mm^2^ at successive depths were performed using the imaging system, Vivastack, which generates multiple confocal images at successive depths at a certain location in the tissue. The methodology for the acquisition of images followed the protocol established by Andrade et al. (52).

### Characterization of the attributes of actinic keratosis through RCM imaging

After the acquisition of the RCM images, the attributes of the scale were defined compiling the different tecidual transformation signals presented in the scientific literature for AK. Further search in the institute’s database allowed us to find different transformation levels for each parameter that were organized exploratory per differentiation level.

The information on the 18 attributes found in the literature, with nomenclature, definitions, and layers where they can be visualized are described in Table 1. The proposed score and its levels are described in Table 2, in which RCM images were gathered on a Photographic Scale, and organized according to 18 of the attributes of cellular transformation with the participant’s samples.

**Table 1:** Microscopic attributes of actinic keratosis.

Attribute number	Name	Definition	Location	References
1	Hyperkeratosis	Thickness of the *stratum corneum* greater than 15 micrometers. Refractable and amorphous structures in the *stratum corneum*	Epidermis, horny layer (*stratum corneum*)	(53, 54)
2	Parakeratosis	Presence of nucleated cells in the *stratum corneum* due to the incomplete keratinization process on the surface of the skin	Epidermis, horny layer (*stratum corneum*)	(53, 54)
3	Desquamation of the *stratum corneum* and corneocyte cohesion	Junction of corneocytes forming scattered plaques. Presence of “chunks” of scattered corneocyte clusters	Epidermis, horny layer (*stratum corneum*)	(55)
4	Cellular atypia in the viable epidermis	Disorder in the shape of the cluster of cells of the *stratum granulosum* and *stratum espinosum*	Epidermis, granulous layer (*stratum granulosum*), and spinous layer (*stratum spinosum*)	(38, 56–60)
5	Dyskeratosis	Presence of rounded nucleated cells in the *stratum espinosum*	Epidermis, spinous layer (*stratum spinosum*)	(54)
6	Inflammatory (dendritic) cells in the epidermis	Presence of rounded and refractile cells, appearing in small groups in the *stratum espinosum*	Epidermis, spinous layer (*stratum spinosum*)	(38, 55, 57)
7	Nuclear pleomorphism in the *stratum espinosum*	Variations in the size and shape of cells and nuclei in the *stratum spinosum*	Epidermis, spinous layer (*stratum spinosum*)	(38)
8	Atypical keratinocytes with follicular coverage	Dark openings with great refraction of surrounding cells	Epidermis, spinous layer (*stratum spinosum*), and basal layer	(61)
9	Poorly defined boundaries between keratinocytes	Irregular connections between keratinocytes	Epidermis, spinous layer (*stratum spinosum*), and basal layer	(56)
10	Inflammatory cells in the basal layer	Presence of small and scattered refractable cells in the basal layer	Epidermis, basal layer	(55)
11	Cellular apoptosis in the viable epidermis	Presence of cell death in spots of the epidermis darkened cytoplasm, and bright nucleus	Epidermis	(38, 55, 57)
12	Fibers around the dermal papillae	Presence of a tangle of fibers and dense material around the papillae. Poorly defined papillae	Dermis, papillary layer	(58)
13	Papillary irregularity	Poorly defined dermal papillae surrounded by dense material	Dermis, papillary layer	(58)
14	Rounding of blood vessels in the papillary dermis	Rounded blood vessels traversing the papillae. Refractive cells = blood cells	Dermis, papillary layer	(38, 62)
15	Melanocytes clustered in the dermis	Bulky cells	Dermis	(58)
16	Solar elastosis	Degradation of elastic and collagen fibers in the skin	Dermis	(54)
17	Brain-like, finger-like appearance	Clusters of rounded, misshapen cells	Dermis	(54)
18	Dermal nests	Cell clusters	Dermis	(54, 58)

**Table 2:** Characterization of actinic keratosis by reflectance confocal microscopy and evaluation methods.

Attribute	Example and grading	What to observe
1—Hyperkeratosis	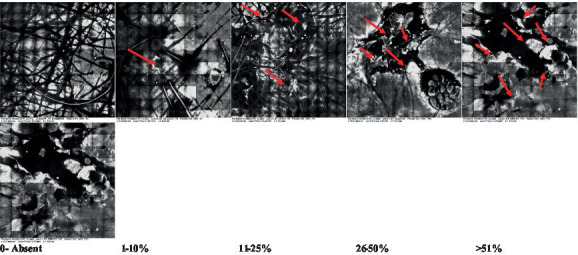	Presence and spreading of visible refractory and amorphous structures in the *stratum corneum* and augmented thickness of the layer
2—Parakeratosis	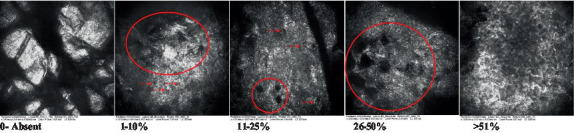	Presence and percentage of spreading area of visible nucleated polygonal cells in the *stratum corneum*
3—Desquamation of the *stratum corneum* and corneocyte cohesion	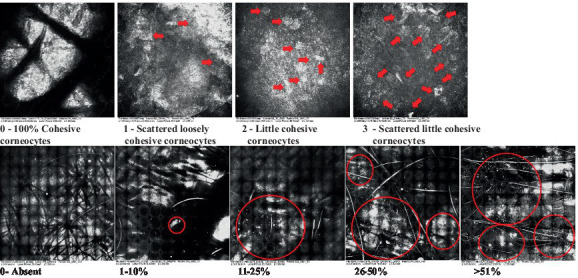	Different levels of cohesiveness of corneocytes and presence and spread of loose horny plates, “chunks” of scattered corneocyte clusters
4—Cellular atypia in the viable epidermis	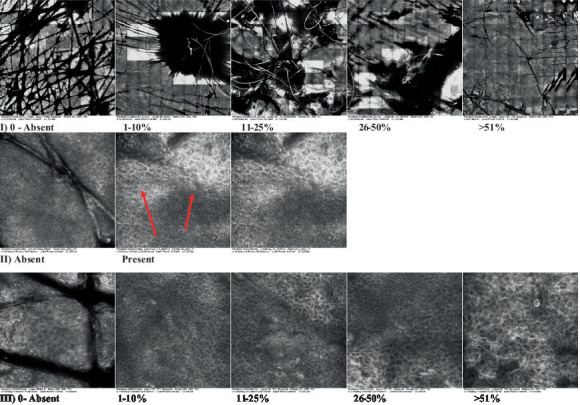	Presence and level of disorder in the shape of the cluster of cells of the viable epidermis (I—atypical honeycomb pattern), *stratum espinosum* (II—disarrangement) and *granulosum* (III—atypical honeycomb pattern), respectively
5—Dyskeratosis	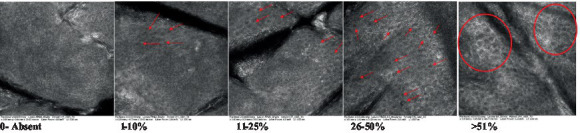	Presence and spreading of rounded nucleated cells in the *stratum espinosum*
6—Inflammatory (dendritic) cells in the epidermis	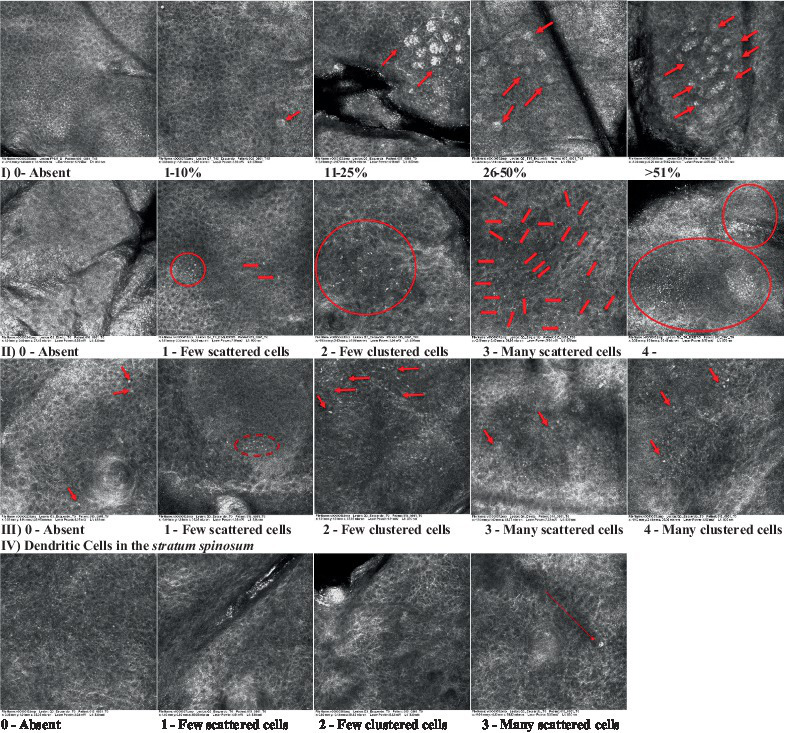	Presence and spreading of rounded and refractile cells, appearing in small groups in the *stratum granulosum* (I—lymphocytes, and II—rounded dendritic cells) and *espinosum* (III—lymphocytes and IV—dendritic Cells), respectively
7—Nuclear pleomorphism in the *stratum spinosum*	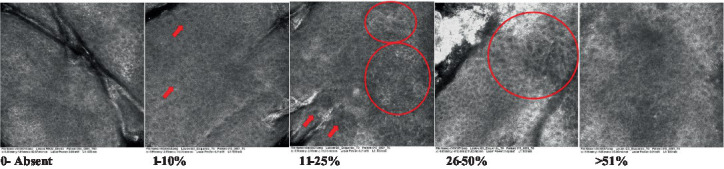	Variations in the size and shape of cells and nuclei in the *stratum spinosum*
8—Atypical keratinocytes with follicular coverage	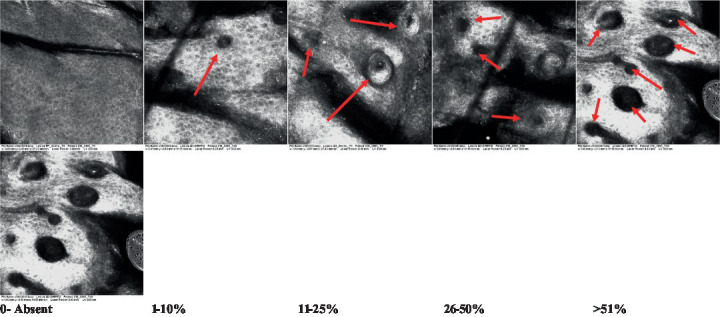	Presence and spreading of dark openings with great refraction of surrounding cells
9—Poorly defined boundaries between keratinocytes	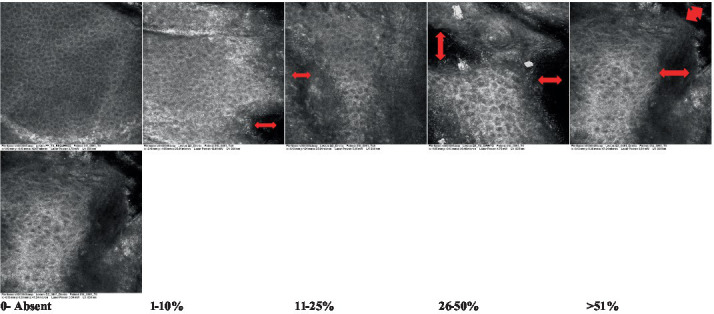	Presence and spreading of Irregular connections between keratinocytes
10—Inflammatory cells in the basal layer	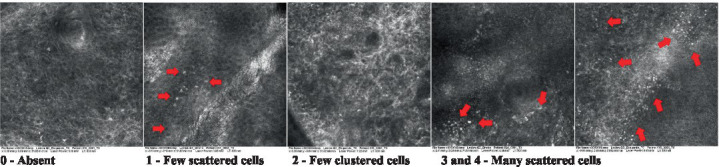	Presence and spreading of small and scattered refractable cells in the basal layer
11—Cellular apoptosis in the viable epidermis	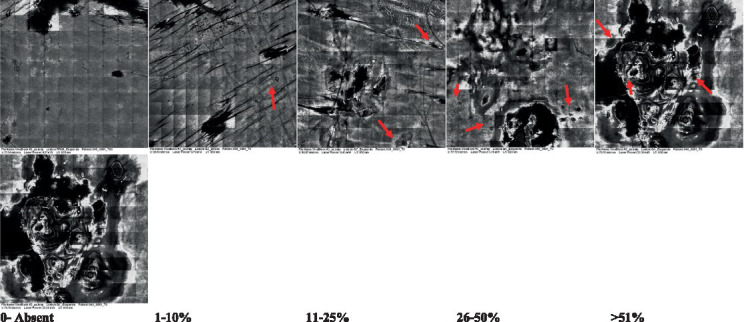	Presence and spreading cell death in spots of the epidermis, darkened cytoplasm and bright nucleus
12—Fibers around the dermal papillae	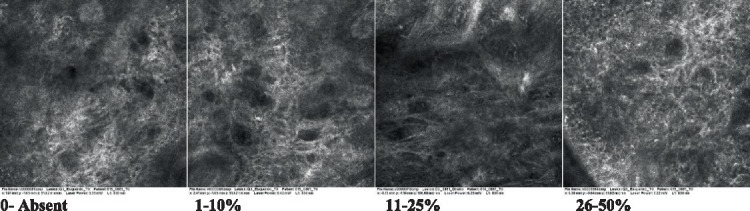	Presence and spreading of entangled fibers and dense material around the papillae. Poorly defined papillae
13—Papillary irregularity	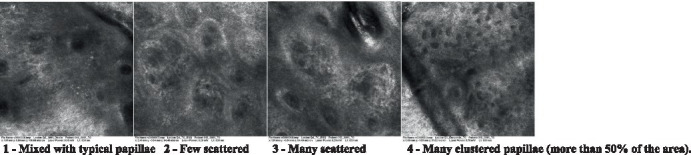	Presence and spreading of poorly defined dermal papillae surrounded by dense material
14—Rounding of blood vessels in the papillary dermis	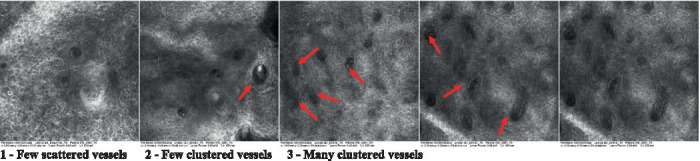	Presence of rounded blood vessels traversing the papillae. Refractive cells = blood cells
15—Melanocytes clustered in the dermis	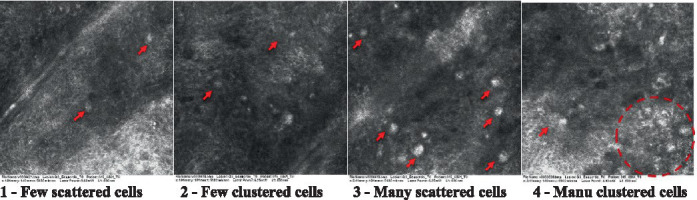	Presence and spreading of bulky melanocytes
16—Solar elastosis	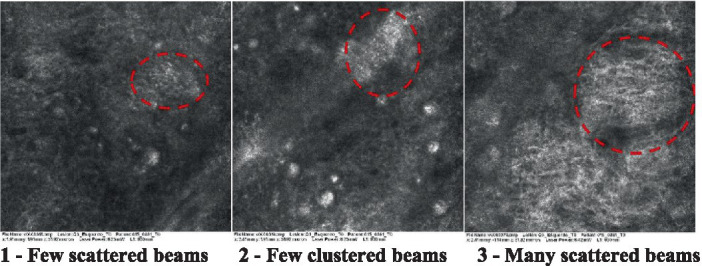	Presence of degraded elastic and collagen fibers in the skin
17—Brain-like, finger-like appearances	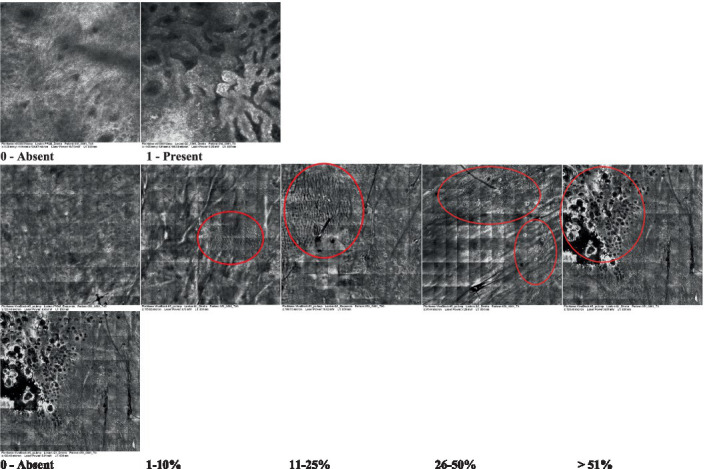	Presence and spreading of clusters of rounded, misshapen cells
18—Dermal nests	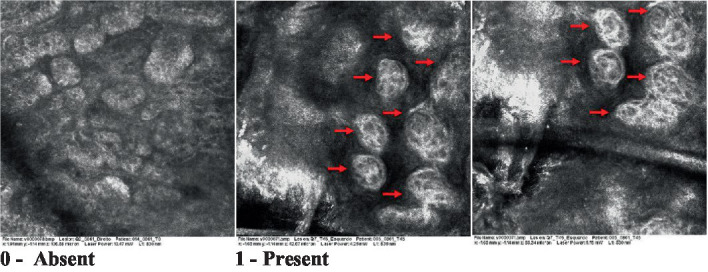	Presence of cell clusters in the dermis

Observations: the presence/absence of each attribute was observed and the relative proportion of the injured area to the total area of the block was calculated. The percentage was then established: 0 for absence; 1–10%; 11–25%; 26–50% and above 50%. Arrows point to individual structures and circles highlight larger regions containing structures. The photos that do not present any highlighted structure are considered the general perspective of the structures present throughout the image.

Scores were given to each characteristic of cellular transformation evidence to classify the type, spreading, and severity of the transformation. The grading system for RCM imaging data was divided into four major categories—individual images, z-stacks, mosaics, and stacks of mosaics (blocks) which correspond to an adaptation of the grading system of histological analysis of keratinocytes neoplasia to a 3D perspective (29).

The score scale varied among attributes, so 3 different scales gathered the 18 attributes evaluated, in which 3 attributes present a scale of 0 or 1; 1 attribute with a scale of 0–3, and, 14 attributes with a scale of 0–4. A total score for the definition of an AK score is also proposed. For this calculation, each evaluated attribute was transformed into a scale ranging from 0 to 100 and a mean was calculated. The higher the score the higher the level of transformation of the evaluated lesion, then, the higher the AK severity.

### Preliminary validation and efficacy study

After the scale was built, two RMC images (stacks) per study subject in each time point (T0 and T90) were scored using the proposed scale by the specialist. Eighteen attributes were evaluated as defined in Table 1, totaling 84 RMC images assessed. A mean score per subject per time-point was calculated through the average score between the two stacks graded.

The reliability of the proposed score on characterizing AK was tested by relating the RMC images score AK obtained by the scale to the visual score AK degree given by a trained Dermatologist. If a correlation were found between the visual diagnosis and the proposed scale, then the scale would be considered efficient in detecting and grading the severity of AK.

To evaluate the repeatability of the method by the grader, all images were scored on two consecutive days (day 1 and day 2), comprising 168 assessments. The repeatability would be achieved if no statistically significant difference were found between the average score of AK on day 1 and day 2.

The scale capability of discrimination between treatments was tested through the clinical preliminary study, to evaluate the performance of the scale in an efficacy study. The results on AK severity after treatment with the topical product containing 0.1% retinoic acid versus the placebo product were assessed using the proposed scale.

### Statistical parameters for the scale validation

Statistical analysis of 21 subjects (10 = product, 11 = placebo) was performed using the software XLSTAT (2023) to compare the intervention groups within the period of treatment.

The validation study had the following analyses carried out: repeatability between days; difference between the group treatments (placebo and treatment); difference between lesion areas and correlation of the given score with clinical grading of AK, variation between the attributes and the differentiation power.

For repeatability between days and the difference between the group treatments (placebo and treatment), an ANOVA model was performed to validate the scoring method along days and the efficacy of the treatment, in which the difference of the total score among time-points (T90 to T0) was considered as the variable response, while day of the assessment (1 or 2), Treatment (product or placebo) and the interaction between of day and treatment were considered as predictor variables.

Each attribute was also compared individually between days and group treatments. However, it was not possible to create a parametric model (ANOVA) for the breakdown for each of the 18 attributes due to discrete responses, which did not accept the normality of the data. Therefore, the non-parametric Kruskal–Wallis test was performed to compare treatments overall per day and between days.

The total score was also correlated to the AK degree through Spearman’s correlation coefficient.

## Results

### Characterization of the attributes of actinic keratosis through RCM imaging

Due to the heterogeneous nature of actinic keratoses, lesions can vary and are difficult to grade. They can occur in all layers of the skin and may appear simultaneously in more than one layer, with no documented appearance pattern (29). The cellular attributes of AK that can be found in literature are organized in Table 1 along with their definitions and the layer in which they can be found. The images of those attributes found in the subjects’ lesions were gathered and evaluated according to the developed grading system and were organized in Table 2.

### Validation study of the photographic scale by evaluating the effectiveness of topical treatment for actinic keratosis

**Figure 1:**
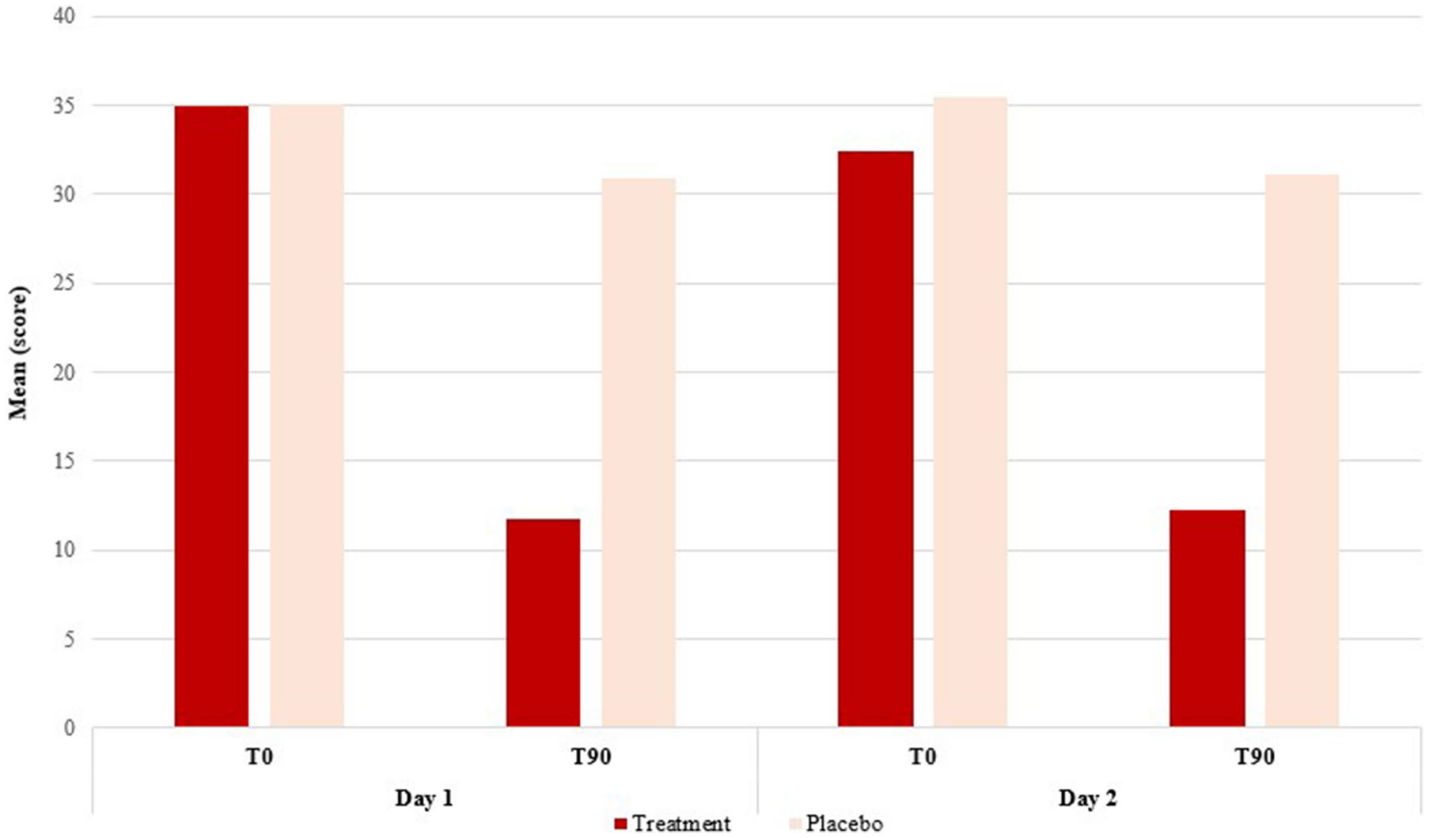
Difference of means between the groups treated with the placebo and with the treatment formulation. Representation of days 1 and 2 of skin analysis at the initial time (T0) and final time (T90), after 90 days of treatment.

For the AK score (mean of transformed attributes per subject), lower scores were observed for the treatment group in T90 (after treatment) when compared to the placebo group performance on both days of analysis (ANOVA, *F* = 33.926; DF = 1.000; *p* = <0.0001), indicating that the proposed score is accurate enough to discriminate among treatments. The result between the days was similar (ANOVA, *F* = 0.241; DF = 1.000; *p* = 0.626) (Figure 1 and Table 3), indicating the repeatability of this method.

**Table 3:** Repeatability of the results of topical treatment.

Source	Degree of freedom	Sum of squares	Mean squares	*F*	*p*-value
Day	1.000	22.667	22.667	0.241	0.626
Treatment	1.000	3187.703	3187.703	33.926	<0.0001
Day × Treatment	1.000	27.078	27.078	0.288	0.595

There was no significant effect of day and treatment day interaction (ANOVA, *F* = 0.288; DF = 1.000; *p* = 0.595), highlighting the repeatability between days.

**Figure 2:**
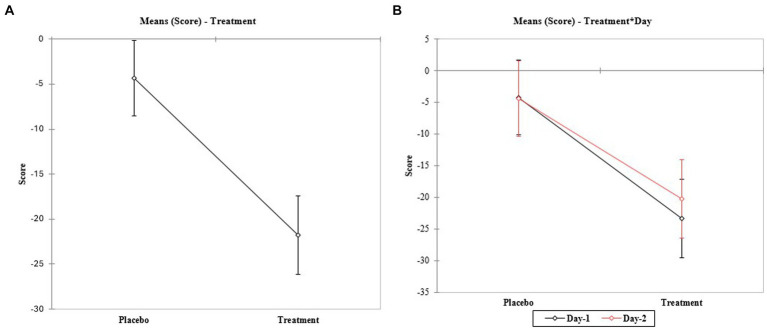
Interaction between days of analysis. **(A)** The comparison of means of interaction between the scores and the treatment groups (placebo and treatment). **(B)** Comparison of means of interaction between the scores and the treatment groups in each day of analysis (day 1 and 2, respectively).

Significant differences were observed between group treatments (Figure 2 and Table 3). When the 18 attributes were evaluated individually, no difference between days was observed for any evaluated attribute (Table 3). Lower scores were observed for the treatment group in T90 (after treatment) when compared to the placebo group performance for 10 out of 18 attributes (Table 4).

**Table 4:** Variation among the transformation attributes.

	*p*-values			
Attribute	Comparison between treatments			
	Day 1	Day 2	General	Comparison between days
1—Hyperkeratosis	0.052	0.131	**0.015**	0.868
2—Parakeratosis	0.064	0.089	**0.012**	0.629
3—Desquamation of the *stratum corneum* and corneocyte cohesion	0.788	0.760	0.681	0.837
4—Cellular atypia in the viable epidermis	0.141	0.618	0.164	0.639
5—Dyskeratosis	**0.000**	**0.001**	**<0.0001**	0.475
6—Inflammatory (dendritic) cells in the epidermis	0.417	0.685	0.384	0.674
7—Nuclear pleomorphism in the *stratum espinosum*	**0.012**	**0.016**	**0.000**	0.902
8—Atypical keratinocytes with follicular coverage	0.287	0.118	0.064	0.978
9—Poorly defined boundaries between keratinocytes	0.064	0.176	**0.021**	0.857
10—Inflammatory cells in the basal layer	**0.031**	**0.034**	**0.002**	0.989
11—Cellular apoptosis in the viable epidermis	0.127	0.487	0.113	0.517
12—Fibers around the dermal papillae	**0.043**	0.143	**0.014**	0.638
13—Papillary irregularity	**0.046**	0.090	**0.009**	0.967
14—Rounding of blood vessels in the papillary dermis	**0.014**	**0.024**	**0.001**	0.645
15—Melanocytes clustered in the dermis	0.099	0.099	**0.021**	0.837
16—Solar elastosis	0.466	0.818	0.501	0.848
18—Dermal Nests	0.942	0.543	0.604	0.672

^*^The attribute 17 (brain-like, finger-like appearance) had all values 0.000, so they were omitted. In bold are the *p*-values <0.05.

### Correlation score versus clinical grading of actinic keratosis

**Figure 3:**
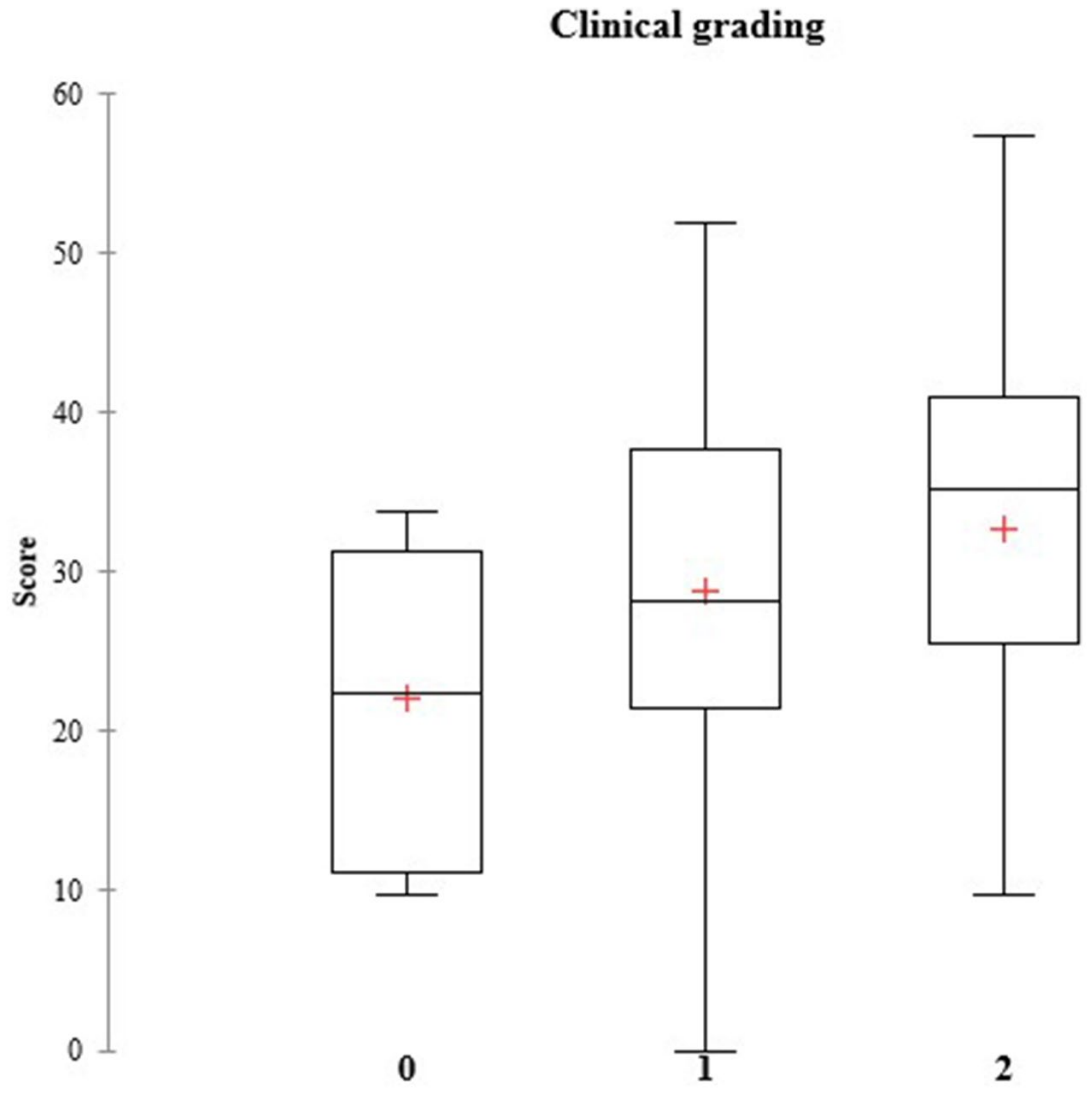
Clinical grading for actinic keratosis. Correlation of the three possible degrees of actinic keratosis (0, 1, and 2) with the scores. The clinical gradings for AK are represented as the following: 0—slightly visible and palpable; 1—visible and palpable, and 2—frankly visible and hyperkeratotic.

There was a statistically significant correlation between the clinical grading of keratosis and the score obtained (Spearman’s *ρ* = 0.229; *p*-value = 0.004), suggesting that the evaluated score is indeed related to the severity of the AK skin lesions (Figure 3 and Table 4).

## Discussion

### Characterization of the attributes of actinic keratosis and correlation score versus clinical grading

Within the study conditions, it was possible to infer that the proposed score developed to grade by imaging the severity and spreading of the subclinical attributes of AK presented a significant correlation with the clinical form of grading AK, suggesting that it represents a consistent and accurate tool, considering that it supports the analysis at the cellular level.

AK is a highly prevalent disease and it is showing a tendency to increase in the following years (17, 18, 24). In AK, the visible lesions are the initial manifestation of a disease continuum that progresses from subclinical keratinocyte dysplasia into invasive cSCC. Its diagnosis must be accurate and once detected it must be treated properly to avoid recurrence or further complications in the treatment and also, cause discomfort to the patient (59).

Nowadays there are many non-invasive and more automated skin assessment techniques such as RCM, having a standardized tool to assist in the diagnosis of keratosis and other pre-cancerous diseases only adds value in clinical practice (63, 64).

Reflectance confocal microscopy (RCM) enables the histological changes that characterize AK to be imaged and graded to be used in the diagnosis and characterization of the disease (40, 65, 66). Also, RCM studies can provide evidence of efficacy for topical treatments, even if the subject only presents subclinical signs of transformation (24). Since RCM is a modern, real-time, and non-invasive method, it is possible to be used together with the scale during all the treatment stages, comparing the patient’s images before, during, and after treatment, in addition to enabling monitoring of treatment in case of recurrence, keeping medical history.

### Validation study of the photographic scale by evaluating the effectiveness of topical treatment for actinic keratosis

The validation study showed that it is possible to evaluate the improvement of the attributes of AK using the scores proposed in the scale since the treatment group presented lower scores when compared to the placebo group on both days of analysis after 90 days of treatment. The data did not present statistical differences between days of analysis, highlighting the repeatability of the scores.

Regarding the treatment chosen for this validation study, the topical treatment with 0.1% retinoic acid showed great improvement as expected. Moreover, the proposed score of the AK lesions was capable of discriminating this improvement.

It is not easy to treat advanced lesions, even with the advances in the area of pharmaceutical sciences, the emergence of nanoemulsions, and the evolution of drug delivery systems, and non-invasive biophysical devices, it is still often necessary to remove the lesion through surgical procedures. When the diagnosis is late, the number of lesions can be so large that their surgical removal is unfeasible. The earlier the diagnosis is given, the easier the treatment is. In this sense, it is much easier to prevent AK than to treat it. Therefore, having a quick diagnosis and characterization of the subclinical attributes and first lesions still in their initial phase will allow faster treatment, with drugs that are less irritating to the skin, and also provide better life quality for the patient. Additionally, Eisen et al. (67) reinforce the good patient-clinician relationship in sharing the decision about the choice of therapy since these decisions will ultimately balance the patient’s compliance habits, ability to tolerate skin reactions or discomfort, duration of therapy, and achieving a successful outcome.

Although these results must be interpreted cautiously due to the small sample size, this study proposed a powerful visual tool, a photographic scale based on RCM—a modern and effective imaging method—the subclinical characteristics of AK, to assist in the identification and characterization of the cellular transformation of AK and, consequently, facilitate early diagnosis, patient monitoring and also a smoother treatment, minimizing both patient discomfort and preventing future lesions and further complications in the cases. The results of the validation study with topical treatment with retinoic acid showed the usability of the scale, comparing the presence and severity (given by the proposed scores) of the 18 defined and detailed attributes with the usual clinical grading given by physicians. Plus, the statistical parameters showed good repeatability, correlation with clinical grading, and differentiation power. These results validate the proposed tool as a quantitative method to evaluate AK.

## Conclusion

AK is a pre-cancer condition that must be accurately diagnosed and treated. RCM is a great tool to provide visual microscopic information on the subclinical stages of AK and to follow the disease’s progression or regression. This study brings a photographic scale to serve as a guide to dermatologists to identify the multiple signs of cellular transformation in a quantitative approach, and consequently, provide early diagnosis, adequate treatment, and follow-up of the lesions throughout the patient’s life. The high resolution and capacity for different treatments also suggest good applicability in comparative clinical trials.

## Data Availability

The raw data supporting the conclusions of this article will be made available by the authors, without undue reservation.
